# Observation of 1,3-Diketones Formation in the Reaction of Bulky Acyl Chlorides with Methyllithium

**DOI:** 10.3390/molecules17066415

**Published:** 2012-05-29

**Authors:** Jian Zhang, Nianfa Yang, Liwen Yang

**Affiliations:** Key Laboratory of Environmentally Friendly Chemistry and Applications of the Ministry of Education, College of Chemistry, Xiangtan University, Xiangtan 411105, China; Email: zhangjian.507@163.com

**Keywords:** acyl chloride, menthylformyl, methyllithium, C_2_-symmetric, 1,3-diketone

## Abstract

The formation of 1,3-diketones was observed in the reactions of bulky acyl chlorides with methyllithium. The reaction products depend on the steric hindrance around the carbonyl group of the acyl chloride and the electronic effect of the group(s) linked to the carbonyl. When the steric hindrance around the carbonyl group of the acyl chloride is big enough, the 1,3-diketone is the only product. In the case of the moderate hindrance around the carbonyl group of the acyl chloride, a moderate yield of 1,3-diketone is obtained and some tertiary alcohol is generated. When there is no steric hindrance around the carbonyl group of the acyl chloride, the tertiary alcohol is the only product. When the steric hindrance around the carbonyl group is moderate and an electron-donating group is connected to the carbonyl of the acyl chloride, all three products—ketone, 1,3-diketone and tertiary alcohol—can be isolated from the reaction mixture after long reaction times.

## 1. Introduction

It is well known that the reaction of acyl chlorides with alkyllithiums produces a tertiary alcohol [1]. A ketone is obtained under mild conditions when the alkyllithium is replaced by lithium dialkylcopper and this reaction provides a convenient method for preparation of ketones with various structures [2]. Recently, we found that 1,3-diketones were obtained when acyl chlorides with large steric hindrance reacted with methyllithium. 1,3-Diketones are important intermediates in organic synthesis [3,4]. They are widely represented in natural products, pharmaceuticals, and other biologically relevant compounds, or are key intermediates for synthesizing such species [5], so the synthesis of 1,3-diketones has attracted much interest from synthetic chemists and many advances have been reported in this area [3]. Recently, Coltart *et al*. reported that ketones undergo soft enolate formation and acylation on treatment with MgBr_2_ OEt_2_, i-Pr_2_NEt, and various acylating agents to give 1,3-diketones [5]. More recently, Kerrigan *et al*. reported the synthesis of 1,3-diketones through ring-opening of ketoketene dimer β-lactones [6]. The discovery of the formation of bulky 1,3-diketones in the reaction of bulky acyl chlorides with methyllithium might provide a supplementary method for the preparation of the 1,3-diketones with bulky groups. Herein we report our work in this area.

## 2. Results and Discussion

### 2.1. The Reaction of Menthylformyl Chloride with Methyllithium

**Scheme 1:**
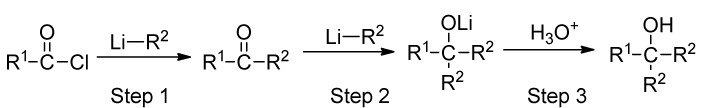
The reaction of an acyl chloride with alkyllithium to generate a tertiary alcohol.

It has been known that the reaction of acyl chlorides with alkyllithiums produces a tertiary alcohol and the reaction is a three-step process as described in Scheme 1. The first step is the substitution of chloride by an alkyl group to form a ketone; the second one is an addition of the alkyllithium to the carbonyl of the formed ketone to generate a tertiary lithium alkoxide and the last one is a hydrolysis of the tertiary lithium alkoxide to produce a tertiary alcohol.

**Figure 1:**
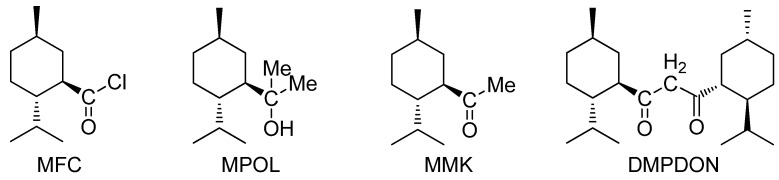
The structures of MFC, MPOL, MMK and DMPDON.

This reaction result has been accepted without question for a long time. However, recently, we discovered that the reaction of menthylformyl chloride (MFC) with methyllithium (MeLi) afforded neither 2-menthyl-2-propanol (MPOL, a tertiary alcohol) nor menthyl methyl ketone (MMK), but 1,3-dimenthylpropane-1,3-dione (DMPDON, a 1,3-diketone, IUPAC name: bis[(1R,2S,5R)-2-isopropyl-5-methylcyclohexyl]propan-1,3-dione) (Figure 1).

**Scheme 2:**
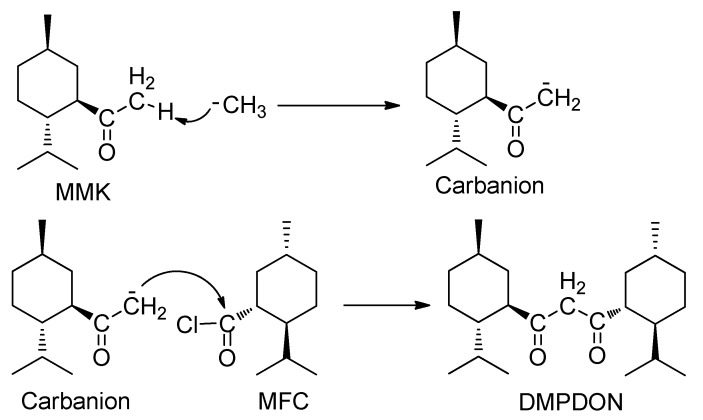
The mechanism of formation of DMPDON.

This result indicated that, after the formation of MMK, MeLi did not attack the carbonyl of MMK but rather abstracted a proton from the methyl connected to the carbonyl to form a menthylformylmethyl carbanion and, subsequently the formed carbanion attacked another MFC to generate DMPDON (Scheme 2).

**Scheme 3:**
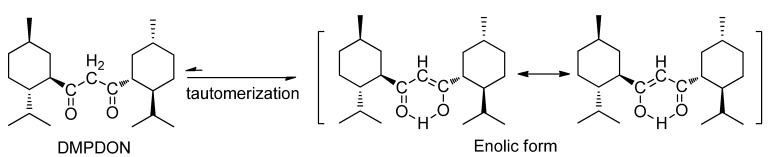
The keto-enol tautomerism of DMPDON.

The reason that the attack of methyl carbanion is at the hydrogen atom of the acylmethyl but not at carbonyl is that there is a large steric hindrance around the latter due to the bulky menthyl group. As is well known, 1,3-diketones bearing an α-proton always exist in the more stable enolic form [3]. The keto-enol tautomerization of DMPDON also takes place as described in Scheme 3.

The structure of enolic DMPDON has been confirmed by MS and NMR. The mass spectrum shows its molecule ion peak at 348.3 (*m/z*). There are 12 carbon signals in its ^13^C-NMR spectrum with a signal at 198.04 ppm which is the characteristic signal of a carbonyl carbon in the enolic form of a 1,3-diketone and a signal at 98.7 ppm, which is the characteristic signal of active methene carbon of the enolic form of a 1,3-diketone. The ^1^H-NMR spectrum shows 18 proton signals with a singlet peak at 15.5 ppm, which is the characteristic signal of a hydroxyl proton in the enolic form of a 1,3-diketone and a singlet peak at 5.49 ppm, which is the characteristic proton signal of the methine between the two carbonyls of the enolic form of a 1,3-diketone. What is interesting is that the 1,3-diketone DMPDON is an optically active, C_2_-symmetrical molecule and there are two bulky chiral groups. These characteristics make DMPDON a potential excellent chiral ligand for the preparation of chiral catalysts.

### 2.2. The Effect of the Reaction Condition on the Yield of 1,3-Dimenthylpropane-1,3-dione

The experiment results of the reaction of MFC with MeLi are listed in Table 1. The reaction temperature has a great influence on the yield of DMPDON. From runs 1–5 in Table 1, we can conclude that the best reaction temperature is 0 °C. When the reaction temperature is higher than 0 °C or lower than 0 °C, the yield of DMPDON will decrease.

**Table 1:** The influence of the reaction temperature on the yield of 1,3-dimenthylpropane-1,3-dione ^a^.

Run	Solvent ^b^	MFC/MeLi ^c^	Temperature (°C)	Yield (%) ^d^
1	Toluene	1/1.1	−78	20.7
2	Toluene	1/1.1	−5	61.3
3	Toluene	1/1.1	0	67.1
4	Toluene	1/1.1	25	40.5
5	Toluene	1/1.1	50	22.6
6	Toluene	1/1	0	65.2
7	Toluene	1/2.1	0	67.0
8	Toluene	1/3.1	0	67.1
9 ^e^	Toluene	1/1.1	0	57.4
10	Tetrahydrofuran	1/1.1	0	62.5
11	Diethyl ether	1/1.1	0	60.2

^a^ MFC: 3.2 mL (1.01 g, 5 mmol), reaction time: 24 h; ^b^ The volume of solvent: 6 mL; ^c^ The mole ratio of MFC to MeLi, MeLi is a solution in diethyl ether (1.6 mol/L); ^d^ The isolated yield; ^e^ 5 mmol of triethylamine was added.

The mole ratio of MFC to MeLi also has a great influence on the yield of DMPDON. The best molar ratio of MFC to MeLi for obtaining a high yield of DMPDON is 1:1.1 (see runs 3, 6 and 7). As mentioned above, MeLi did not attack the carbonyl of the intermediate MMK to form a tertiary alcohol due to the big steric hindrance of the menthyl group. Once MMK is formed, another MeLi molecule immediately abstracted the α-proton at the methyl of MMK to form a carbanion and the carbanion attacked another MFC molecules to form a 1,3-diketone. In the whole process, 1 mole of MFC consumed 1 mole of MeLi. When the mole ratio of MFC to MeLi was less than 1:1.1, any increase of the quantity of MeLi had no significant influence on the yield of DMPDON (cf. run 3 with run 7 and run 8 of Table 1), indicating that the MMK forming step is a rate-determining step. In fact, some menthylformic acid was found in the waste water from the work-up process at the end of the reaction, indicating that reaction of MFC was incomplete within 24 h. The addition of triethylamine decreases the yield of DMPDON (run 9 in Table 1). The nature of the solvent has a moderate influence on the yield of DMPDON. Among the three tested solvents (toluene, tetrahydrofuran and diethyl ether), toluene is the best one (see runs 3, 10, and 11 in Table 1).

### 2.3. The Effect of the Bulk of Group Linking to Carbonyl of Acyl Chloride on the Reaction

**Scheme 4:**
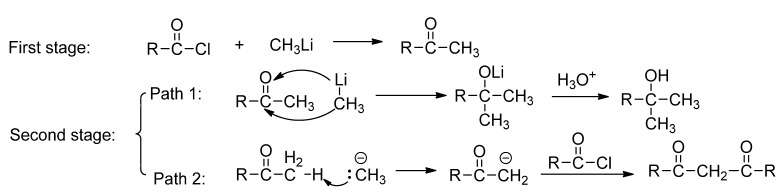
The reaction of an acyl chloride with methyllithium.

The reaction of an acyl chloride with MeLi consists of two stages as described in Scheme 4. In the first stage, the acyl chloride reacted with MeLi to form a methyl ketone. Then, in the second stage, the nucleophilic MeLi attacked the carbonyl of the intermediate methyl ketone to give a tertiary alcohol (Path 1 in Scheme 4) or abstracted a proton from the methyl of the produced methyl ketone to form an enolic anion, which continued to attack another acyl chloride to give a 1,3-diketone (Path 2 in Scheme 4).

To gain insight into the reaction of acyl chlorides with methyllithium and examine whether this method is applicable for preparation of other 1,3-diketones, several different acyl chlorides were used to replace MFC. The results are listed in Table 2.

**Table 2:** The reaction of acyl chlorides with methyllithium ^a^. 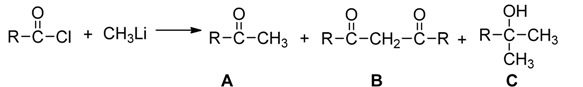

Run	R	Yield/% ^b^		
		A	B	C
1	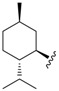	-	67.0	-
2	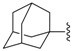	-	34.0	36.1
3 ^c^	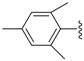	7.9	49.9	21.6
4	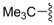	-	10.2	84.1
5 ^d^	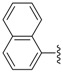	-	-	91.1
6	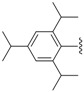	69.4	12.2	-

^a^ Acyl chloride: 5 mmol, the mole ratio of acyl chloride/MeLi = 1/1.1, solvent: 6 mL of toluene, reaction temperature: 0 °C, reaction time: 24 h; ^b^ The isolated yield; ^c^ reaction time: 72 h; ^d^ Acyl chloride: 5 mmol, the mole ratio of acyl chloride/MeLi was 1/2.1.

Under the experimental conditions, acyl chlorides with different structures gave different results. Menthylformyl chloride gave only 1,3-diketone in 67% isolated yield due to its big steric hindrance. Adamantylformyl chloride gave 1,3-diketone with the isolated yield being 34%, with 36.1% yield of tertiary alcohol being obtained because the steric hindrance was not large enough to block the addition of another MeLi molecule to the carbonyl. In the case of 2,4,6-trimethylbenzoyl chloride, after reacting for 72 h, the isolated yield of 1,3-diketone is 49.9%, and 7.9% yield of ketone and 21.6% yield of tertiary alcohol were obtained. The reason why there was still some ketone remaining was that the acylmethyl proton on the ketone might be unfavorable for abstraction by another MeLi molecule due to the presence of the electron-donating 2,4,6-trimethylphenyl group and that the carbonyl might be very sterically obstructed for the addition of another MeLi molecule to it. 2,4,6-Triisopropylbenzoyl chloride gave mainly methyl ketone, with the isolated yield being 69.4%, along with a 12.2% yield of 1,3-diketone being obtained (run 7 in Table 2). After the three methyl groups of 2,4,6-trimethylbenzoyl chloride were replaced by isopropyl groups, the steric hindrance around the carbonyl increased tremendously so that the attack of MeLi at the carbonyl was blocked completely and no tertiary alcohol was produced. The steric hindrance of two isopropyl groups at the 2-position and 6-position was so tremendous that the reaction of the formed methyl ketone with another 2,4,6-triisopropylbenzoyl chloride was hampered. Another factor which disturbs the formation of the 1,3-bis(2,4,6-triisopropylphenyl)-1,3-propanedione was the electron-donating property of the three isopropyl groups. tert-Butylformyl chloride gave a little diketone (10.2% isolated yield) and a large amount of tertiary alcohol (84.1% isolated yield) due to its smaller steric hindrance round the carbonyl group. 1-Naphthylformyl gave only tertiary alcohol and no 1,3-diketone was isolated because there was almost no steric hindrance around the carbonyl group.

## 3. Experimental

### 3.1. Instruments and Materials

^1^H-NMR and ^13^C-NMR spectra were recorded on a Bruker Avance 400 spectrometer at 400 MHz and 100 MHz, respectively, using CDCl_3_ as a solvent and tetramethylsilane as an internal standard. Optical rotations (OR) were measured using a Perkin Elmer 341 LC polarimeter. Toluene, tetrahydrofuran and diethyl ether were refluxed with sodium and benzophenone and distilled before use. Menthylformyl chloride was prepared from (−)-menthol according to the method reported by Wu and Yang [7]. Other chlorides were prepared from the corresponding carboxylic acids using thionyl chloride at reflux temperature as chlorination reagent. Other reagents were commercially available reagents without special treatment.

### 3.2. The Typical Reaction Process

An argon-flushed flask equipped with a dropping funnel and stirrer was charged with solvent (3 mL) and a desired amount of methyllithium solution in diethyl ether. The flask was placed in a cooling bath. acyl chloride (5 mmol) dissolved in solvent (3 mL) was added dropwise into the flask with stirring at desired temperature. The mixture was stirred at the same temperature for 24 h after the addition. The reaction was quenched with ice water. The organic layer was separated and the aqueous layer was extracted with diethyl ether. The organic layer and extracts were combined. The combined solution was dried over anhydrous sodium sulfate and, then, was concentrated under reduced pressure. The concentrated residue was chromatographed on silica gel to obtain products.

### 3.3. Chromatographic Eluents and Characterization of the Products

*1,3-Bis[(1R,2S,5R)-2-isopropyl-5-methylcyclohexyl]propan-1,3-dione* (DMPDON). Chromatography solvent: 1:20 dichloromethane/petroleum ether; yellow crystals, m.p. 88–90 °C; [α]20 D = −200 (c = 2.0, THF); MS: 348.3 *m/z*; Elem. Anal. calcd. for C_23_H_40_O_2_: C, 79.25; H, 11.57. found: C, 79.21; H, 11.61; ^1^H-NMR δ/ppm: 0.76 (d, *J* = 6.8 Hz, 6H, 2 × CH_3_), 0.88~0.91 (m, 12H, 4 × CH_3_), 0.96~2.10 (m, 18H, 6 × CH_2_, 6 × CH), 2.13 (dt, *J* = 11.4 Hz, *J* = 3.1 Hz, 2H, CH_2_CHCO) 5.49 (s, 1H, CH=OH), 15.85 (s, 1H, CH=OH); ^13^C-NMR δ/ppm for the enolic form: 15.96, 21.24, 22.30, 24.16, 28.71, 32.42, 34.75, 39.75, 44.56, 50.77, 98.67, 198.02.

*1,3-Bis(tricyclo[3.3.1.13,7]dec-1-yl)propan-1,3-dione*. Chromatography solvent: 1:10 dichloromethane/petroleum ether; pale yellow crystals, m.p. 271–273 °C (lit. [8]: 268–270 °C); Elem. Anal. calcd. for C_23_H_32_O_2_: C, 81.13; H, 9.47. found: C, 81.16; H, 9.45; ^1^H-NMR δ/ppm: 1.67 (m, 12H, 6 × CH2), 1.72 (brs, 12H, 6 × CH2), 2.00 (brs, 6H, 6 × CH), 5.66 (s, 1H, CH=OH), 16.37 (s, 1H, CH=OH); ^13^C-NMR δ/ppm: 18.50, 28.75, 37.06, 37.46, 41.04, 107.40, 154.70.

*2-(Tricyclo[3.3.1.13,7]dec-1-yl)-2-propanol*. Chromatography solvent: 1:10 dichloromethane/petroleum ether; yellow crystals, m.p. 73–75 °C (lit. [9]: 77 °C); Elem. Anal. calcd. for C_13_H_22_O: C, 80.35; H, 11.41. found: C, 80.36; H, 11.39; ^1^H-NMR δ/ppm: 1.16 (s, 6H, CH_3_), 1.65~1.73 (m, 13H, adamantane-H, -OH), 2.02 (brs, 3H, 3 × CH); ^13^C-NMR δ/ppm: 24.24, 28.65, 36.26, 37.13, 38.75, 41.23, 74.77.

*1,3-Bis(2,4,6-trimethylphenyl)propan-1,3-dione*. Chromatography solvent: 1:2 dichloromethane/petroleum ether; white crystals, m.p. 106–108 °C (lit. [10]: 103–104 °C); Elem. Anal. calcd. for C_21_H_24_O_2_: C, 81.78; H, 7.84. found: C, 81.79; H, 7.82; ^1^H-NMR δ/ppm: 2.32 (s, 18H, CH_3_), 5.75 (s, 1H, CH=OH), 6.88 (s, 4H, CH, phenyl), 16.05 (s, 1H, CH=OH); ^13^C-NMR δ/ppm: 19.54, 21.07, 105.47, 128.69, 134.50, 134.77, 138.90, 191.18.

*2,4,6-Trimethylacetophenone*. Chromatography solvent: 1:2 dichloromethane/petroleum ether; pale yellow liquid; Elem. Anal. calcd. for C_11_H_14_O: C, 81.44; H, 8.70. found: C, 81.40; H, 8.72; ^1^H-NMR δ/ppm: 2.23 (s, 6H, PhCH_3_), 2.29 (s, 3H, PhCH_3_), 2.47 (s, 3H, COCH_3_), 6.85 (s, 2H, CH, phenyl); ^13^C-NMR δ/ppm: 19.12, 21.10, 32.21, 128.53, 132.35, 138.33, 139.97, 208.48 (the NMR data is the same as in the literature [11]).

*2-(2,4,6-Trimethylphenyl)-2-propanol*. Chromatography solvent: 1:2 dichloromethane/petroleum ether; yellow crystals, m.p. 113–115 °C (lit. [12]: 110–111 °C); Elem. Anal. calcd. for C_12_H_18_O: C, 80.85; H, 10.18. found: C, 80.86; H, 10.19; ^1^H-NMR δ/ppm: 1.68 (s, 7H, C(CH_3_)_2_OH), 2.21 (s, 3H, CH_3_, p-phenyl), 2.44 (s, 6H, CH_3_, o-phenyl), 6.75 (s, 2H, CH, phenyl); ^13^C-NMR δ/ppm: 20.20, 25.82, 33.81, 56.09, 132.30, 134.87, 136.20, 143.00.

*2,2,6,6-Tetramethylheptan-3,5-dione*. Chromatography solvent: 1:10 dichloromethane/petroleum ether; pale yellow liquid; Elem. Anal. calcd. for C_11_H_20_O_2_: C, 71.70; H, 10.94; found: C, 71.73; H, 10.92. ^1^H-NMR δ/ppm: 1.26 (s, 18H, CH_3_), 5.75 (s, 1H, CH=OH), 16.19 (s, 1H, CH=OH); ^13^C-NMR δ/ppm: 25.20, 39.88, 90.71, 201.47. (The NMR data is the same as the literature [13]).

*1,1,2,2-Tetramethyl-2-propanol*. Chromatography solvent: 1:10 dichloromethane/petroleum ether; colorless liquid, b.p. 132 °C (760 mm Hg); Elem. Anal. calcd. for C_7_H_16_O: C, 72.35; H, 13.88; found: C, 72.36; H, 13.89. ^1^H-NMR δ/ppm: 0.96 (s, 9H, (CH_3_)_3_C); 1.19 (s, 6H, t-BuCCH_3_); ^13^C-NMR δ/ppm: 25.32, 25.39, 46.21, 77.56. (The NMR data is the same as the literature [14]).

*1-Naphthyl-2-propanol*. Chromatography solvent: 1:5 dichloromethane/petroleum ether; white crystals, m.p. 83–85 °C (lit. [15]: 83–85 °C); Elem. Anal. calcd. for C_13_H_14_O: C, 88.83; H, 7.58. found: C, 88.86; H, 7.54. ^1^H-NMR δ/ppm: 1.88 (s, 7H, CH_3_, OH), 7.38~7.52 (m, 3H, Ar-H), 7.59 (d, *J* = 7.1 Hz, 1H, Ar-H), 7.78 (d, 1H, *J* = 8.1 Hz, Ar-H), 7.88 (d, *J* = 7.3 Hz, 1H, Ar-H), 8.83 (d, *J* = 8.2 Hz, 1H, Ar-H); ^13^C-NMR δ/ppm: 31.68, 74.13, 122.69, 124.82, 125.29, 125.70, 126.46, 128.36, 128.62, 131.94, 134.99, 143.51.

*2,4,6-Triisopropylacetophenone*. Chromatography solvent: 1:5 dichloromethane/petroleum ether; white crystals, m.p. 83–85 °C (lit. [16]: 84 °C); Elem. Anal. calcd. for C_1__7_H_26_O: C, 82.87; H, 10.64. found: C, 82.86; H, 10.63. ^1^H-NMR δ/ppm: 1.22~1.25 (m, 18H, CHCH_3_), 2.29 (s, 3H, COCH_3_), 2.70~2.73 (m, 3H, CHCH_3_), 7.00 (s, 2H, Ar-H); ^13^C-NMR δ/ppm: 23.94, 24.34, 31.01, 33.86, 34.32, 121.03, 138.45, 143.18, 149.41, 209.16.

*1,3-Bis(2,4,6-triisopropylphenyl)propane-1,3-dione*. Chromatography solvent: 1:5 dichloromethane/petroleum ether; yellow crystals, m.p. 92–93 °C; MS: 476.4 *m/z*; Elem. Anal. calcd. for C_33_H_48_O_2_: C, 83.14; H, 10.15. found: C, 83.12; H, 10.16. ^1^H-NMR δ/ppm: 1.16 (d, 24H, *J* = 6.4 Hz, CHCH_3_), 1.27 (m, 14H, COCH_2_CO, CHCH_3_), 2.64 (m, 4H, CHCH_3_), 2.91 (m, 2H, CHCH_3_), 7.05 (s, 4H, Ar-H); ^13^C-NMR δ/ppm: 23.86, 23.99, 29.69 (CO**C**H_2_CO), 31.98, 34.42, 121.17, 132.33, 145.91, 150.91, 200.86.

## 4. Conclusions

The reaction products of acyl chlorides with methyllithium depend on the steric hindrance around the carbonyl group of the acyl chloride and the electronic effects of the group(s) connected to the carbonyl. When the steric hindrance around the carbonyl group of the acyl chloride is big enough, a 1,3-diketone is the only product and this provides a simple, effective method for the preparation of bulky C_2_-symmetric 1,3-diketones which are excellent potential ligands for the preparation of chiral catalysts. In the case of the moderate hindrance around the carbonyl group of the acyl chloride, a moderate yield of 1,3-diketone forms, along with some tertiary alcohol. When there is no steric hindrance around the carbonyl group of the acyl chloride, tertiary alcohol is the only product. When the steric hindrance around the carbonyl group is moderate and an electron-donating group is connected to the carbonyl of the acyl chloride, all the three products—ketone, 1,3-diketone and tertiary alcohol—can be isolated from the reaction mixture after long reaction times.
